# Changes on the Caco-2 Secretome through Differentiation Analyzed by 2-D Differential In-Gel Electrophoresis (DIGE)

**DOI:** 10.3390/ijms131114401

**Published:** 2012-11-07

**Authors:** Andrés García-Lorenzo, Ana M. Rodríguez-Piñeiro, Francisco J. Rodríguez-Berrocal, María Páez de la Cadena, Vicenta S. Martínez-Zorzano

**Affiliations:** Department of Biochemistry, Genetics and Immunology, Faculty of Biology, University of Vigo, 36310 Vigo, Spain; E-Mails: andres_galo@uvigo.es (A.G.-L.); berrocal@uvigo.es (F.J.R.-B.); mpaez@uvigo.es (M.P.C.)

**Keywords:** Caco-2, secretome, differentiation, DIGE, colorectal cancer, biomarkers

## Abstract

Colorectal cancer is still a major health burden worldwide, and its diagnosis has not improved in recent years due to a lack of appropriate diagnostic serum markers. Aiming to find new diagnostic proteins, we applied the proteomic DIGE technology to analyze changes in the secretome before/after differentiation of the colon adenocarcinoma Caco-2 cell line, an accepted *in vitro* model to study colorectal tumorigenesis. When the secretomes from undifferentiated (tumor-like) and differentiated cells (resembling healthy enterocytes) were compared, we found 96 spots differentially expressed. After MS/MS analysis, 22 spots corresponding to 15 different proteins were identified. Principal component analysis demonstrated these 22 spots could serve as a discriminatory panel between the tumor-like and normal-like cells. Among the identified proteins, the translationally-controlled tumor protein (TCTP), the transforming growth factor-beta-induced protein ig-h3 (TGFβIp), and the Niemann-Pick disease type C2 protein (NPC2) are interesting candidates for future studies focused on their utility as serum biomarkers of colorectal cancer.

## 1. Introduction

Colorectal cancer (CRC) represents globally the third leading cause of cancer-related mortality (after lung and breast cancer) [1], being one major health burden. Its development is a multi-step process that usually spans about 10 years, which should give an opportunity for early detection [2]. However, CRC diagnosis is usually made when cancer has spread to adjacent tissues due to its lack of symptoms and appropriate screening markers, since most of the molecules in use (CEA, CA-19.9, CA-72.4) show low specificity and/or sensitivity for the disease. Therefore, the finding of new and useful biomarkers for CRC would be of great relevance if early detection and even prevention could be achieved, especially through screening of a healthy population.

As one of the characteristics of an ideal tumor marker should be its tissue specificity, we aimed to find molecules produced in epithelial intestinal cells and secreted or shed into the surrounding tissue or the circulation. Therefore, we studied the secretome of the Caco-2 cells. This cell line was established from a moderately well-differentiated human colon adenocarcinoma. When cultured *in vitro* over confluence under standard culture conditions, it spontaneously differentiates into a cell type with remarkable intestinal enterocyte-like features, including brush borders with microvilli on their apical side, tight junctions, and enterocytic hydrolase activities [3–6]. This differentiation process provides a valuable research tool, as undifferentiated Caco-2 cells resemble those found in tumor tissues, whereas the differentiated ones lose the tumorigenic phenotype and are similar to healthy enterocytes [7]. Therefore, proteins found differently expressed in between those two cell types are firm candidates for further explorations in human healthy and tumor colorectal tissues. The subset of proteins occurring in conditioned media from cultured cells is defined as the “secretome” of those cells [8]. These proteins released by tumor cells *in vitro* may, to a certain extent, reflect the proteins released by tumors *in vivo* (*i.e.*, the cancer secretome). Thus, analysis of tumor cell-derived secretomes may represent a feasible strategy for finding potential serum biomarkers for cancer [9,10].

The use of proteomics methods to analyze the secretome of Caco-2 cells before and after differentiation allows searching for a panel of potential biomarkers, instead of only one or few proteins, eventually enhancing both the sensitivity and specificity of the disease detection. In particular, for this study we chose an adaptation of the two-dimensional electrophoresis (2-DE) called differential in-gel electrophoresis (DIGE). This technique not only allows the separation of proteins on the basis of their isoelectric point and relative molecular mass, but to run up to two different samples and an internal standard in the same gel. The method is based on the differential resolution of the cyanine dyes (Cy2 or cyanine, Cy3 or indocarbocyanine, and Cy5 or indodicarbocyanine), covalently linked to lysine residues on the proteins. These fluorescent dyes have all similar charge and mass, and only a minimal amount is used to label the sample [11,12], hence they produce a very small and reproducible modification on the protein mass; besides, they compensate the original positive charge on the lysine residue bound with their own quaternary ammonium. Therefore, proteins will migrate mostly according to its original mass and net charge. The method has been described to have a sensitivity as low as 0.1 ng [13]. When an internal standard (pool of all samples analyzed, usually labeled with Cy2) is added the method becomes quantitative, as protein abundances can be normalized [11]. It also permits automated spot detection and alignment, decreasing the number of operator-related errors. Therefore, DIGE shows high sensitivity and reproducibility, with a wide dynamic range.

The DIGE technology has been already used in numerous studies. In CRC research there are several early examples as the work of Friedman *et al.*[14], who detected 52 proteins characteristic of tumor tissues, or the study of Alfonso *et al.*[15], where the authors found 41 proteins altered in tumor tissues and related to such important processes as transcription, cellular communication, or signal transduction. In our group, DIGE was applied to serum samples from CRC patients and healthy donors, in order to find potential biomarkers for the pathology [16]. DIGE has been also applied to *in vitro* studies with different cell lines, among them to the cellular proteome of Caco-2 cells [17–19]. This has been also compared between proliferative and differentiated states through methodologies other than DIGE. In particular, the nucleus [20] and the plasma membrane [21] of those cells were compared to find proteins related with the differentiation process. Recently, a whole-cell approach was taken, and a comparison between the proteome of proliferating and differentiated cells revealed 53 proteins with differential regulation [6]. Interestingly, this study showed an upregulation in non-differentiated Caco-2 cells of proteins involved in cell growth or proliferation, and related to cancer, confirming the tumoral phenotype of these cells. Previous transcriptomic analyses had already shown that proliferating Caco-2 cells resemble cancer cells, whereas the differentiated phenotype was suitable as a model of the intestinal barrier [22,23].

Regarding the secretome of human CRC cell lines, it was studied in Smad4-deficient and Smad4-re-expressing derivatives of the SW480 cell line, finding more than 25 differential proteins including chaperones, proteases and protease inhibitors [24]. In a study of the cell lines Colo205 and SW480, the collapsin response mediator protein-2 was chosen as a potential CRC biomarker [9]. On the other hand, comparison of the primary cell line SW480 and its metastatic derivative SW620 yielded 145 differential proteins, from which the trefoil factor 3 and the growth/differentiation factor 15 were validated as potential biomarkers for CRC metastasis [25].

However, to date nobody has investigated the changes along the differentiation process induced on the proteins secreted by the Caco-2 cells. Therefore, we thought worth to study their secretome, as proteins secreted/shed by the undifferentiated cells, but not by the differentiated ones, could also be secreted/shed by intestinal tumor cells into the circulation after losing their “normal” phenotypes. This approach could identify a set of proteins that are potentially interesting serum biomarkers of the intestinal malignant transformation.

## 2. Results and Discussion

### 2.1. Differentiation of the Caco-2 Cell Line

In order to study the secretome of undifferentiated and differentiated cells, Caco-2 cell cultures were set up and allowed to differentiate as described before [3]. This was done in several parallel flasks, and repeated three times. The main advantage of this model, when compared with direct plasma or serum analyses, is that here only what the malignant cell (undifferentiated) or its counterpart (differentiated) secretes to the medium is detected, while in serum/plasma studies many detected proteins may not have been secreted by the malignant cells. On the other hand, the main drawback of this system is that cultured cells are serum-starved for 24 h, which induces stress on cells that become prone to spontaneous autolysis, resulting in non-specific release of intracellular proteins [26]. This could obscure the interpretation of results, though this phenomenon should affect both culture types and thus the non-specifically released proteins may not be detected as differential between them.

**Figure 1:**
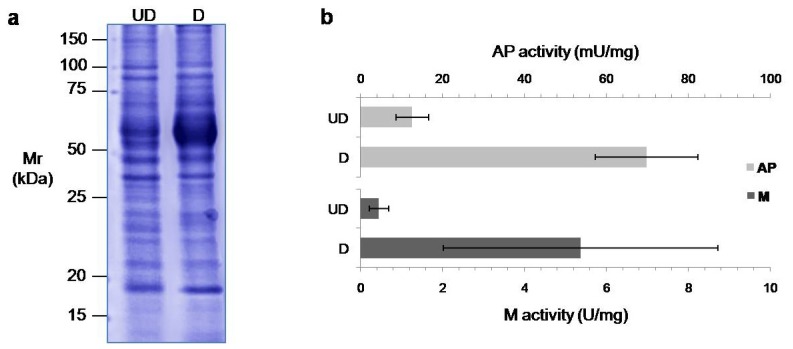
(**a**) Monodimensional protein pattern detected by Coomassie staining of the secretome of undifferentiated (UD, left) and differentiated (D, right) Caco-2 cells; Mr: relative molecular mass; (**b**) Specific activity of the enzymes alkaline phosphatase (AP, clear bars) and maltase (M, dark bars), known markers of enterocytic differentiation.

In the three experiments, we counted an average of 7.4 ± 1.6 million cells in the undifferentiated flasks, and 10.6 ± 2.2 million cells in the differentiated ones. When the secretome of these cells was obtained, the amount of protein recovered was 196.4 ± 10.4 μg for the undifferentiated cells, and 262.9 ± 17.1 μg for the differentiated ones. The secretome was visualized for both types of cells by monodimensional electrophoresis (Figure 1a) which demonstrates some differences in the secreted proteins patterns between undifferentiated and differentiated Caco-2 cells. To corroborate the differentiation process, we measured the specific activities of the enterocytic enzymes alkaline phosphatase and maltase after differentiation in three independent experiments, observing an average 6-fold and 14-fold increase, respectively (Figure 1b).

### 2.2. Comparison of the Secretome of Undifferentiated and Differentiated Caco-2 Cells

**Figure 2:**
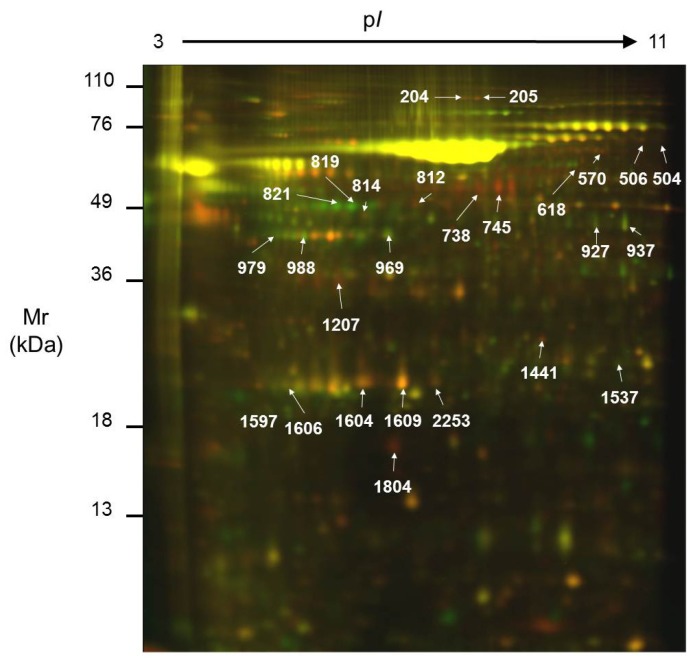
Representative Differential In-Gel Electrophoresis (DIGE) analytical gel where the secretome of an undifferentiated Caco-2 culture has been labeled with Cy5 (red spots) and the secretome from a differentiated culture with Cy3 (green spots). Yellow areas represent overlapping spots. The spots identified are labeled with the spot number. Mr: relative molecular mass; pI: isoelectric point.

The DIGE technique was used for the analysis of the secretome of Caco-2 cells, before and after differentiation, in the three independent experiments. Following the DIGE experimental design, we combined one undifferentiated and one differentiated sample, plus a pooled internal standard, per gel. Therefore, we analyzed nine samples in three gels. In Figure 2, we show a representative image from one such gel. The samples were randomly distributed as stated in Table 1.

After acquiring the three images of each gel, the protein spots were detected and aligned by comparison with the internal standard. The number of spots detected and matched in each gel is shown in Table 1. More than 1600 distinct protein spots were detected in each gel. This result is in agreement with those described for other CRC cell lines. As an example, more than 1000 distinct spots have been detected in the secretome of SW480 cells [24], whereas the secretome of the cell line LIM1215 allowed detection of approximately 2000 spots by DIGE [27].

**Figure 3:**
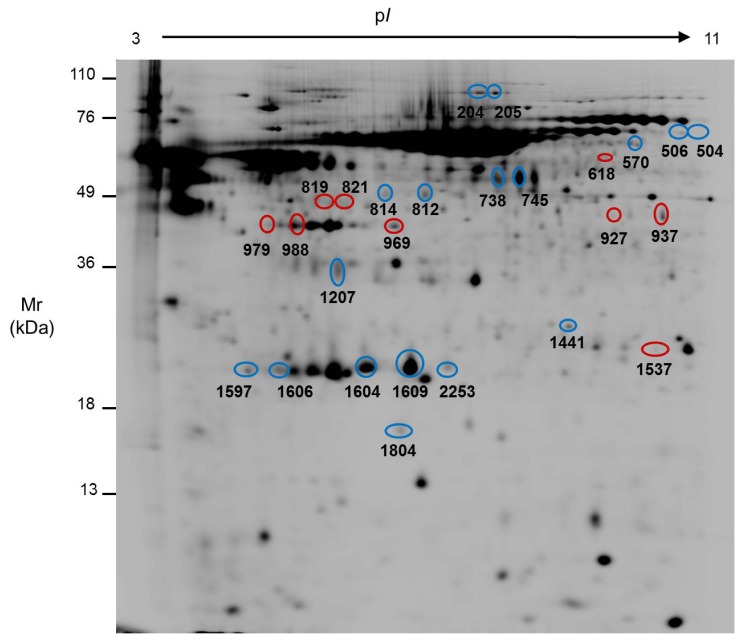
Representative 2-D map where the spots with significant variation due to Caco-2 differentiation identified by MS are highlighted. The spot numbers correspond to those shown in Table 2. Blue circles indicate higher expression and red circles lower expression in undifferentiated (tumor-like) cells. Mr: relative molecular mass; pI: isoelectric point.

For the quantitative comparison, the abundance of each spot was made relative to the total amount of protein secreted by the number of live cells in the corresponding sample, and only the 919 spots detected in all the samples were considered. Applying the Mann-Whitney U test, we found 96 spots with significantly different abundance (*p* ≤ 0.05) in the secretome of Caco-2 undifferentiated and differentiated cells. From those spots, we were able to locate and cut 34 out of a preparative gel, and those were analyzed by mass spectrometry. Eventually, we identified the 26 spots highlighted in Figures 2 and 3, and summarized in Table 2. Four of them were identified as potential contaminants, and therefore not included in Table 2 (spot 812: mixture of human cytokeratin 1, keratin 9 and type I keratin 16; spots 819 and 821: bovine albumin; spot 1606: bovine apolipoprotein A–I). As an example of the variation in abundance, some of the identified spots are shown in Figure S1, where 3-D images have been drawn on the basis of the spot abundance after normalization. Noticeably, some of the proteins were identified in several different spots, probably due to their bearing different post-translational modifications.

**Figure 4:**
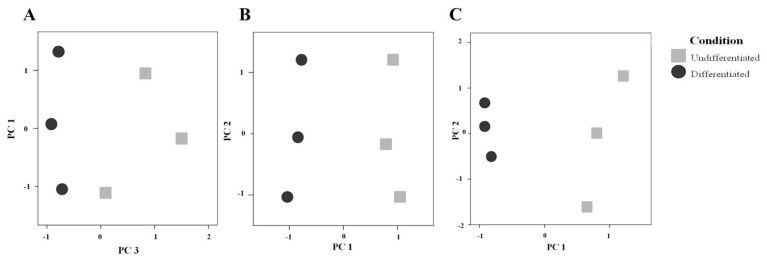
(**a**) Plot of the most relevant principal components (PCs) found when analyzing the abundance of all the protein spots detected by DIGE in the secretome of undifferentiated and differentiated Caco-2 cells. These cell states can be differentiated by PC3 (X axis), reflecting a characteristic protein pattern before and after differentiation. The *Y* axis (PC1; same results found for PC2) groups the samples on the basis of the gel were they ran, indicating a technical contribution; (**b**) Separation given by principal component analysis (PCA) only on the spots that were significantly different between the cell states; (**c**) Separation of cell states given by PCA only on the 22 identified spots.

Finally, we applied the multivariate test PCA on the spot abundances. When PCA was applied to the 919 spots detected in all samples, it allowed the discrimination of the secretome of the undifferentiated cells from that of the differentiated cells. The first three principal components (PCs) explained 85.5% of the variability between groups; PC3 was able to distinguish day 6 and day 20 samples (*p* ≤ 0.05 by Mann-Whitney U test) (Figure 4a). Interestingly, both PC1 and PC2 could separate the samples per gel, that is, samples run on the same gel obtained similar values for those PCs. This highlights the importance of using an internal standard to allow matching between different gels, and thus correcting for technical variations. PC1 and PC2 accounted for 70% of the variance, whereas PC3, responsible for the difference between sample groups, accounted for 15.5% of the variance. When the PCA was repeated with only the 96 spots found significantly altered after differentiation, we found that the first PC was significant and explained by 64% of the variance of the data, giving a neat separation between the cell states, as shown in Figure 4b. Even with the 22 identified spots, the first PC was significant and was able to explain 65% of the variance, separating samples from both undifferentiated and differentiated secretome, as shown in Figure 4c.

Among the proteins differently secreted by the Caco-2 cells before/after differentiation, we found enzymes involved in the glycoprotein metabolism (glucosidase II), carbohydrate metabolism (triose phosphate isomerase, NADP-dependent isocitrate dehydrogenase), energy metabolism (ATP synthase subunit beta, creatine kinase B), and detoxification (glutathione *S*-transferase omega-1), as well as lipid transport proteins (apolipoproteins A-I and A-IV, apolipoprotein E precursor) and proteins involved in hemostasis/tissue homeostasis (fibrinogen gamma chain). Considering the functions developed by those proteins, and the fact that alterations in their expression could be related to different physiological situations (as inflammation, starvation, exercise, *etc.*), they are likely to show low specificity as CRC biomarkers. Nevertheless, it is interesting to notice some of these proteins had been related before to the differentiation of Caco-2 cells, in particular by Stierum *et al.*[5] during the comparison of cellular lysates of Caco-2 cells before and after differentiation. In the case of the ATP synthase beta, these authors could not determine the direction of the change, while we found a higher amount (6.8 ratio) of the secreted form in undifferentiated cells. For the gluthathione *S*-transferase, Stierum *et al.* observed an up-regulation of the cytoplasmic form of the glutathione *S*-transferase A1, while we detected the form omega-1 with a 1.6 times higher amount in the secretome of undifferentiated cells. Higher secretion in the undifferentiated cells could be compatible with a smaller amount of protein retained in the cytoplasm in these cells, and therefore with increased intracellular expression in the differentiated cell lysates, as Stierum *et al.* reported. Another protein detected by these authors was the creatine kinase B, for which we found a higher amount of secretion in differentiated cells (ratio undifferentiated/differentiated = 0.3), while Stierum *et al.* found an upregulation followed by downregulation as the cells became differentiated. Finally, the triose phosphate isomerase was also identified in the lysates analyzed by Stierum *et al.*, though its change was not determined. In our study, we found higher secretion of this enzyme in the differentiated cells.

In contrast to the proteins mentioned above, the translationally-controlled tumor protein, the transforming growth factor-beta-induced protein ig-h3, and the Niemann-Pick disease type C2 protein, seem to be the most interesting candidates as potential CRC biomarkers.

The translationally-controlled tumor protein (TCTP) was identified from one spot overexpressed in the secretome of undifferentiated cells, showing an average increase of 3-fold. This protein was first described in mouse tumor cells as a growth-related protein [28]. Despite its name, it is a highly conserved and ubiquitously expressed protein in all eukaryotes, highlighting its important role in the cell [29]. Although the TCTP functions are not well defined, previous studies demonstrated it is implicated in histamine release [30], calcium binding [31], and that it is anti-apoptotic [32]. Nowadays TCTP is described as a multifunctional protein that plays important roles in cell proliferation, immune response, tumorigenicity, and cell death, including apoptosis [29]. TCTP is secreted by different cell types, as macrophages [33] or human embryonic kidney cells [34]. Regarding CRC, the level of TCTP mRNA detected in three human colon carcinoma cell lines (SNUC2A, SNU-C4, and SNU-C5) suggests that a high TCTP mRNA expression in these cells could be related to the rapid cell growth and, therefore, a high potential of tumorigenesis [35]. Moreover, knockdown of TCTP inhibited proliferation, migration, and invasion activities in human colon adenocarcinoma LoVo cells [36]. Related to the cell line used on this work, Stierum *et al.*[5] observed the same trend on TCTP expression when comparing undifferentiated and differentiated Caco-2 cell lysates as we have now found in their secretome.

The transforming growth factor-beta-induced protein ig-h3 (TGFβIp) was identified from three different spots, all of them overexpressed in the undifferentiated Caco-2 cells. These different isoforms could be due to post-translational modifications, since at least 29 sites of vitamin K-dependent carboxylation have been described and annotated for this protein (http://www.uniprot.org/uniprot/Q15582), as well as several phosphorylation sites (http://www.phosphosite.org/proteinAction.do?id=3567117). TGFβIp, originally known as βig-h3, was first cloned as a TGF-β-induced gene on a human lung adenocarcinoma cell line [37]. It localizes to chromosome 5 (5q31) and consists of 17 exons that span around 34 kb [38]. The protein has 683 amino acids and contains a secretory signal. TGFβIp is not only induced by TGF-β, and other factors may be involved on its regulation. It appears in the extracellular matrix associated with collagen, fibronectin, laminin and glycosaminoglycans, and it supports the adhesion of many cell types by recruiting integrins [39]. This protein has been described both increased and decreased in tumors, but in particular it has shown an increased expression in CRC [40–42]. TGFβIp could be involved in multiple aspects of tumorigenesis, including tumor progression, angiogenesis and metastasis, though its role is not yet clear. In colon cancer it has been demonstrated that ectopic expression of TGFβIp enhanced the aggressiveness and altered the metastatic properties of colon cancer cells *in vivo*, whereas inhibition of its expression dramatically reduced metastasis. Mechanistically, it appears to promote extravasation, a critical step in the metastatic dissemination of cancer cells [43]. Besides, TGFβIp levels on serum have been found to be elevated in pancreatic cancer [44].

The Niemann-Pick disease type C2 protein (NPC2) appeared as one spot with a 6-fold average elevation in the secretome of undifferentiated cells. NPC2 is a cholesterol-binding glycoprotein whose encoding gene is located in chromosome 14 [45]. It consists of a 151-amino acid sequence, and the mature protein has a molecular weight between 17 kDa and 20 kDa. NPC2 is highly conserved among major species, and it is present in fluids such as milk, epididymal fluid, bile, and plasma [46–48]. NPC2 binds cholesterol with high affinity and was implicated in mediating intracellular cholesterol trafficking through the late endosomal/lysosomal compartment [49]. In addition, it has been shown that this protein plays an important role in the regulation of hematopoiesis [50] and immunity [51]. Recently, its implication in adipocyte differentiation [52] and fibroblast activation in disease pathogenesis [53] has been also described. To date there are no reports about any relationship with CRC, but an increased expression of the mRNA of this protein has been described in melanoma [54].

Noticeably, the proteins shown in Table 2 are either annotated in curated databases with a signal peptide, or can be predicted by computational methods to be secreted through non-classical pathways. The only exceptions are two proteins which nonetheless have been described in secretions by other authors. In particular, the creatine kinase B has been described in exosome-like vesicles released by intestinal epithelial cells [55] and in the conditioned medium of prostate cancer cell lines [56], whereas the glutathione *S*-transferase omega-1 has been described in the secretome of different cell lines including the colon cancer cells SW480 and SW620 [25].

Overall, the strategy we describe in the present study serves as a method for identifying proteins specifically altered in relation to colon tumorigenesis, and further studies are needed to validate their utility in serum as biomarkers for the disease.

## 3. Experimental Section

### 3.1. Cell Culture and Enterocytic Differentiation

The Caco-2 human colon adenocarcinoma cell line was purchased from the European Collection of Cell Cultures (ECACC No: 86010202). For routine growth we cultured them in 25 or 75 cm^2^ plastic flasks containing Dulbecco’s modified Eagle’s medium (DMEM) supplemented with 4.5 g/L glucose, 20% fetal calf serum (FCS), 100 units/mL of penicillin, 100 μg/mL of streptomycin, 2 mM l-glutamine, and 1% non-essential amino acids at 37 °C, in a 5%-CO_2_ humidified atmosphere. Cells were sub-cultured after reaching 90% confluence by treating them with a solution of 0.05% Trypsin/0.02% EDTA in PBS. The number of cells per flask was measured by counting them on a Neubauer chamber. Trypan blue was used as a stain to distinguish between dead and living cells.

For differentiation experiments, pre-confluent cultures were disrupted with trypsin, cells were seeded on 75 cm^2^ flasks at 12,000 cells/cm^2^. Medium was changed every day after 48 h of seeding. Considering the descriptions of Pinto *et al.*[3] and Rousset *et al.*[4], and the values of differentiation marker enzymes, we selected day 6 as the undifferentiated state and day 20 as the differentiated one.

### 3.2. Enzymatic Assays

The process of enterocytic differentiation was evaluated through the assay of marker enzymes as the alkaline phosphatase and the maltase. For these assays, cells were harvested by scraping, washed with PBS, pelleted and re-suspended in ice-cold ultrapure water with protease inhibitors (Complete Mini tablets, Roche). Re-suspended pellets were placed on ice for 5 min, homogenized with a Potter-Elvehjem, and then frozen at −80 °C.

The alkaline phosphatase activity (EC 3.1.3.1) was measured with the method described by Engstrom [57]. As substrate we used 16 mM para-nitrophenyl phosphate in pH 9.8 50 mM sodium borax buffer plus 1 M MgCl_2_; 200 μL of substrate were mixed with 50 μL of sample and incubated at 37 °C for 30 min. The reaction was stopped by adding 0.6 mL of 0.25 M NaOH. The mixture was centrifuged at 700 g for 5 min and the optical density (OD) at 410 nm was measured.

The maltase activity (EC 3.2.1.20) was measured following the method of Dahlqvist [58], where maltose served as the substrate. For the assay, 40 μL of sample where incubated with 40 μL of 55 mM maltose in 0.1 M maleate buffer pH 6.0 for 1 h at 37 °C. The glucose generated from the maltose was measured using a commercial enzymatic test (Spinreact) based on the method described by Trinder [59,60].

Protein concentrations were determined using the Bradford microassay [61] and results were expressed as specific activity.

### 3.3. Secretome Collection and Preparation

To study the extracellular medium, cells were kept in FCS-free medium for 24 h before collection [62]. After 24 h, medium was collected and centrifuged at 600 g for 5 min, so that particles on suspension were discarded with the pellet. The medium was then dialyzed for 24 h against ultrapure water (cut-off: 12–14 kDa), freeze-dried on a Christ Alpha 2–4 equipment and kept at −80 °C.

### 3.4. SDS-PAGE

For monodimensional electrophoresis, 10 μg of total protein were separated in 10% (*v*/*v*) polyacrylamide (30% T, 2.6% C) denaturing minigels [63]. Gels were stained with Coomassie brilliant blue for protein visualization.

### 3.5. Differential In-Gel Electrophoresis (DIGE)

Dried medium was reconstituted on ultrapure water and cleaned using 2-D CleanUp Kit (GE-Healthcare). Pellets were diluted on lysis buffer (8 M urea, 2 M thiourea, 4% (*w*/*v*) CHAPS and 30 mM Tris) and the protein concentration was measured using a modified Bradford method [64]. An internal standard was prepared by pooling equivalent amounts of protein from each of the six samples included in the experiment. Stock solutions for the cyanine dyes were reconstituted in *N*,*N*′-dimethylformamide (DMF) keeping the proportion CyDye:DMF at 1:1.5 to obtain the working solution. Cy2 was used for labeling the internal standard, while Cy3 and Cy5 were randomized between undifferentiated and differentiated samples (Table 1). For each sample, 50 μg of protein were labeled with 200 pmol of the correspondent Cy dye in ice for 30 min. Labeling was terminated by adding 1 μL of 10 mM lysine and incubating 10 min in ice.

For the 2-DE separation, a mixture of the Cy2-, Cy3- and Cy5-labeled samples (150 μg), was applied on pH 3–11, 24-cm non-linear Immobiline Dry Strips (GE Healthcare) by cup loading. Isoelectric focusing (IEF) followed this program: 1 h at 120 V, 2 h at 500 V, 2 h at 1000 V, 16 h at 5000 V, and a final holding step of 500 V for no longer than 10 h using the Ettan IPGphor (GE Healthcare). After IEF, strips were equilibrated for 12 min with 6 M urea, 100 mM Tris, 30% (*w*/*v*) glycerol, 2% (*w*/*v*) SDS and 0.5% (*w*/*v*) DTT; then for 6 min with 6 M urea, 100 mM Tris, 30% (*w*/*v*) glycerol, 2% (*w*/*v*) SDS and 4.5% (*w*/*v*) IAA. The second dimension was run on 10% polyacrylamide gels (30% T; 2.6% C) for 17 h at 2 W/gel.

### 3.6. Image Acquisition and Computer Analysis of Electrophoretic Patterns

Three images per gel were acquired with the Typhoon 4900 Imager (GE Healthcare) with the appropriate excitation and emission wavelengths for each of the three dyes. Protein patterns for each sample were then analyzed with the software SameSpots (GE Healthcare). Abundances were given as normalized intensity values corrected by a factor consistent in the total protein quantity of the secretome (μg) divided by the number of cells (in millions) in it, which allowed us to compare the amount of protein secreted by each cell instead of just its proportion in the total pool of proteins.

### 3.7. Statistical and Computational Methods

In order to choose the spots which expression showed significant abundance variation, we applied the non-parametric Mann-Whitney U test. Multivariate studies were done through principal component analysis (PCA) as described before [65]. All analyses were performed with SPSS (release 16) and *p* values ≤ 0.05 were considered statistically significant.

For the proteins identified, we investigated if they had a classical signal peptide annotated in www.uniprot.org. Besides, we employed the Secretome 2.0 Server to predict secretion through a non-classical route [66].

### 3.8. Protein Identification by Mass Spectrometry

Selected spots were cut out from Coomassie-stained gels, reduced, alkylated and in-gel digested with 10 μg/mL trypsin in 25 mM ammonium bicarbonate at 37 °C overnight. Peptides were eluted with 5% (*v*/*v*) trifluoroacetic acid and 75% (*v*/*v*) acetonitrile. Samples were mixed with a matrix of 5 mg/mL α-cyano-4-hydroxycinnamic acid in 50% (*v*/*v*) acetonitrile. The mixture was analyzed by matrix-assisted laser desorption/ionization-time of flight (MALDI-TOF), in a 4800 Proteomics Analyzer (Applied Biosystems) by peptide mass fingerprinting (PMF). When proteins were not identified by this method, peptides were further fragmented (MALDI-TOF/TOF) and analyzed by sequence query (SQ). Database searching was done with the MASCOT Daemon search engine (Matrix Science) against the human nrNCBI database. Search parameters were stated as follows: enzyme trypsin; allowance of 1 missed cleavage; propionamide as fixed modification; methionine oxidation as variable modification; 50 ppm of peptide mass tolerance for PMF; 100 ppm peptide mass tolerance and 0.3 Da fragment mass tolerance for SQ. Protein hits were considered significant when the protein score was above the *p* ≤ 0.05 threshold, with at least two significant peptides.

## 4. Conclusions

Although gel-based proteomic techniques are being rapidly superseded by MS methods, the DIGE technology is still a powerful tool for comparative quantitative studies. In this work, it was applied to the analysis of the secretome of Caco-2 cells resembling tumoral and healthy enterocytes. From the 96 proteins differentially expressed in the tumor-like cells, we could identify 15 proteins, among which TCTP, TGFβIp and NPC2 seem to be promising candidates for serum studies of CRC detection. Further work will investigate whether these proteins show different levels in the serum of healthy individuals and CRC patients in different stages of the disease, probing the utility of these molecules as diagnostic markers for CRC.

## Supplementary Information



## Figures and Tables

**Table 1:** Number of protein spots detected in the three replicates of undifferentiated and differentiated Caco-2 cells.

Gel	No. Spots Detected	No. Spots Matched	* Label	Sample
Gel 1	2254	2254	Cy2	Standard
			Cy5	UD1
			Cy3	D3

Gel 2	1632	1164	Cy2	Standard
			Cy5	UD2
			Cy3	D1

Gel 3	2023	1463	Cy2	Standard
			Cy5	D2
			Cy3	UD3

* Number of spots matched to gel 1, used as template as it contains the higher number of spots; Sample: UD, undifferentiated; D, differentiated.

**Table 2:** Proteins that showed a significant variation in the Caco-2 secretome associated with the differentiation process.

Spot No.	ID	Name	pI	Mr (kDa)	Int. UD (mean ± SD)	Int. D (mean ± SD)	Ratio	Function	Localization	Method	Protein Score	Threshold	Coverage (%)
204	Q14697	Glucosidase II subunit alpha	5.7	107.2	184.36 ± 113.39	61.32 ± 17.73	3.0	*N*-glycoprotein metabolism	ER; Pred. sec.	PMF	249	66	34
205	Q14697	Glucosidase II subunit alpha	5.7	107.2	126.37 ± 61.35	32.18 ± 7.26	3.9	*N*-glycoprotein metabolism	ER; Pred. sec.	PMF	275	66	37
504	Q15582	Transforming growth factor-betainduced protein ig-h3	7.7	74.7	30.31 ± 3.72	15.50 ± 2.20	2.0	Cellular adhesion	Secreted	SQ	107	66	21
506	Q15582	Transforming growth factor-betainduced protein ig-h3	7.7	74.7	65.28 ± 17.57	31.04 ± 3.54	2.1	Cellular adhesion	Secreted	PMF	77	66	25
570	Q15582	Transforming growth factor-betainduced protein ig-h3	7.7	74.7	60.17 ± 10.52	22.14 ± 6.58	2.7	Cellular adhesion	Secreted	SQ	211	56	29
618	P23141	Liver carboxylesterase1	5.9	56.5	24.86 ± 10.16	58.72 ± 19.93	0.4	Detoxification	ER; Pred. sec.	PMF	91	66	34
738	P10619	Cathepsin A	6.0	51.9	959.27 ± 685.01	229.14 ± 142.67	4.2	Carboxipeptidase	Lysosome; Pred. sec.	SQ	104	66	16
745	P06576	ATP synthase subunit beta	5.3	56.5	2445.77 ± 2596.37	360.55 ± 231.07	6.8	ATP synthesis	Mitochondria; Pred. sec.	SQ	115	56	27
814	P02679	Fibrinogen gamma chain	5.5	46.8	141.99 ± 34.59	54.12 ± 23.49	2.6	Hemostasis/acute phase response	Secreted	SQ	97	66	14
927	Q6FHQ6	NADPdependent isocitrate dehydrogenase	6.5	46.9	80.32 ± 6.07	201.39 ± 91.20	0.4	Carbohydrate metabolism	Cytosol; Pred. Sec.	SQ	224	66	23
937	Q6FHQ6	NADPdependent isocitrate dehydrogenase	6.5	46.9	242.59 ± 91.94	569.91 ± 255.38	0.4	Carbohydrate metabolism	Cytosol; Pred. Sec.	PMF	205	66	50
969	P12277	Creatine kinase B	5.3	42.9	253.51 ± 103.76	918.55 ± 495.43	0.3	Energy metabolism	Cytosol	PMF	252	66	56
979	P06727	Apolipoprotein A-IV	5.3	45.4	62.39 ± 32.05	321.76 ± 235.62	0.2	Lipid transport	Secreted	PMF	215	66	49
988	P06727	Apolipoprotein A-IV	5.3	45.4	206.98 ± 52.20	723.07 ± 303.91	0.3	Lipid transport	Secreted	SQ	130	66	34
1207	P02649	Apolipoprotein E precursor	5.7	36.3	354.49 ± 32.66	167.01 ± 85.65	2.1	Lipid transport	Secreted	PMF	215	66	60
1441	P78417	Glutathione *S*-transferase omega-1	6.2	27.8	103.79 ± 13.36	65.54 ± 33.38	1.6	Glutathione Transference	Cytosol	SQ	89	56	17
1537	P60174	Triose phosphate isomerase	6.5	26.8	63.43 ± 22.22	162.21 ± 95.92	0.4	Carbohydrate metabolism	Cytosol; Pred. sec.	PMF	141	66	60
1597	Q5W0H4	Translationallycontrolled tumor protein	5.1	22.8	152.49 ± 38.04	45.49 ± 8.91	3.4	Calcium binding and microtubule stabilization	Cytosol; Pred. sec.	SQ	82	66	35
1604	P02647	Apolipoprotein A-I	5.5	28.9	1935.86 ± 793.28	943.66 ± 494.74	2.1	Lipid transport	Secreted	PMF	191	66	67
1609	P02647	Apolipoprotein A-I	5.5	28.9	4306.10 ± 2751.70	1567.04 ± 913.34	2.8	Lipid transport	Secreted	PMF	270	66	81
1804	P61916	Niemann-Pick disease type C2 protein	5.3	16.6	437.13 ± 260.61	68.25 ± 49.70	6.4	Cholesterol binding	Secreted	SQ	101	66	30
2253	P02647	Apolipoprotein A-I	5.5	28.9	302.18 ± 178.79	118.77 ± 34.88	2.5	Lipid transport	Secreted	PMF	68	66	37

ID: accession number in UniProt; pI: theoretical isoelectric point; Mr: theoretical relative molecular mass; Int: intensity; UD: undifferentiated; D: differentiated; Ratio: average abundance in undifferentiated over differentiated cells; ER: endoplasmic reticulum; Pred. Sec.: Potentially secreted proteins, either containing a classical signal peptide, or predicted by the Secretome 2.0 Server (http://www.cbs.dtu.dk/services/SecretomeP/) to be secreted via a non-classical route; PMF: peptide mass fingerprint; SQ: sequence query; Threshold: lowest protein score value for a positive protein identification (*p* = 0.05); Coverage: percentage of the protein sequence covered by the assigned peptides.
