# Supplementary material for: Changes on the Caco-2 Secretome through Differentiation Analyzed by 2-D Differential In-Gel Electrophoresis (DIGE)
Source: Int J Mol Sci. 2012 Nov 7;13(11):14401–20. doi: 10.3390/ijms131114401 (PMC3509587; doi:10.3390/ijms131114401)
Supplement: Supplementary file 1 [file ijms-13-14401-s001.pdf]

Supplementary Information

Figure S1. Three-dimensional plot of the abundance of some of the spots identified, before (left) and after (right) differentiation.

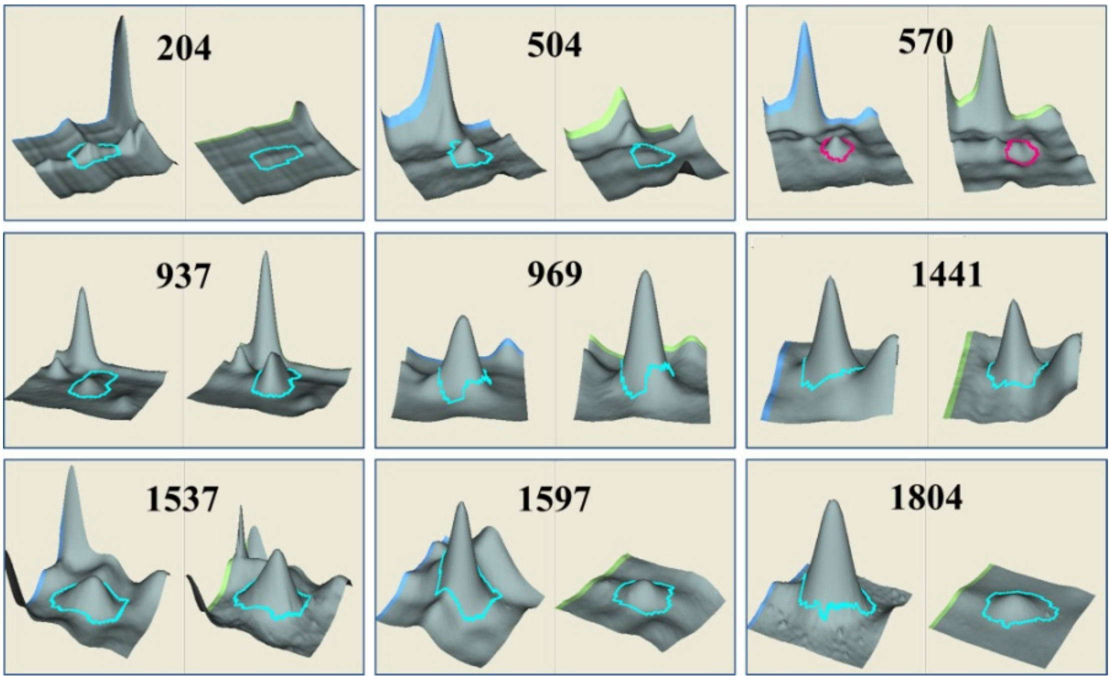

© 2012 by the authors; licensee MDPI, Basel, Switzerland. This article is an open access article distributed under the terms and conditions of the Creative Commons Attribution license (http://creativecommons.org/licenses/by/3.0/).
